# A High-Resolution Demodulation Algorithm for FBG-FP Static-Strain Sensors Based on the Hilbert Transform and Cross Third-Order Cumulant 

**DOI:** 10.3390/s150509928

**Published:** 2015-04-27

**Authors:** Wenzhu Huang, Tengkun Zhen, Wentao Zhang, Fusheng Zhang, Fang Li

**Affiliations:** 1 Optoelectronic System Laboratory, Institute of Semiconductors, Chinese Academy of Sciences, Beijing 100083, China; E-Mails: hwzhu@semi.ac.cn (W.H.); tkzhen@semi.ac.cn (T.Z.); lifang@semi.ac.cn (F.L); 2 Institute of Electrical and Electronic Engineering, Shijiazhuang Tiedao University, Shijiazhuang 050043, China; E-Mail: zhangfusheng020@sina.com

**Keywords:** FBG-based Fabry-Perot interferometers, static strain, demodulation algorithm, Hilbert transform, cross third-order cumulant, high resolution

## Abstract

Static strain can be detected by measuring a cross-correlation of reflection spectra from two fiber Bragg gratings (FBGs). However, the static-strain measurement resolution is limited by the dominant Gaussian noise source when using this traditional method. This paper presents a novel static-strain demodulation algorithm for FBG-based Fabry-Perot interferometers (FBG-FPs). The Hilbert transform is proposed for changing the Gaussian distribution of the two FBG-FPs’ reflection spectra, and a cross third-order cumulant is used to use the results of the Hilbert transform and get a group of noise-vanished signals which can be used to accurately calculate the wavelength difference of the two FBG-FPs. The benefit by these processes is that Gaussian noise in the spectra can be suppressed completely in theory and a higher resolution can be reached. In order to verify the precision and flexibility of this algorithm, a detailed theory model and a simulation analysis are given, and an experiment is implemented. As a result, a static-strain resolution of 0.9 nε under laboratory environment condition is achieved, showing a higher resolution than the traditional cross-correlation method.

## 1. Introduction

In recent years, FBG-based Fabry-Perot interferometers (FBG-FPs) formed by two identical FBGs have been used for many applications in several fields, such as deformation/strain monitoring, vibration measurement, temperature sensing, *etc.* [1,2,3]. FBG-FPs have many obvious advantages owing to their capability of real-time, *in situ*, sensitive strain measurement with low cost, fast response and immunity to electromagnetic interference [4,5]. Especially, high-finesse FBG-FP can be applied to ultra-high-resolution static-strain sensing where an extra FBG-FP is used as a reference sensor head [6,7,8]. In these systems, a narrow line-width tunable laser is generally used for interrogating the FBG-FP sensor heads and the reference FBG-FP is used for compensating any temperature disturbances and laser frequency drift. Then the static strain can be demodulated by calculating the wavelength difference of a group of harmonic peaks from the two FBG-FPs’ reflection spectra in a free spectrum range (FSR).

There are many methods to calculate the wavelength difference of two FBG sensors, such as centroid detection algorithm (CDA) [9], the least square method (LSQ) [10], the autocorrelation [11] and cross-correlation method [12] and so on. Among these algorithms, the cross-correlation algorithm exhibits good ability of suppressing random uncertainty and can realize higher-resolution static-strain measurements [6,7,8,12]. In our previous reports, we have addressed applying the wavelet transform to cross-correlation processing of noise-contaminated FBG-FP reflection spectra for further improving static-strain measurement resolution [13,14]. However, a lot of noise interference in the sensing signals has become the main limiting factor for improving static-strain resolution. These noises include thermal noise, shot-effect noise, laser relative intensity noise, laser frequency noise and other random noise from experimental environment. Most of them, such as thermal noise, shot-effect noise and other random vibration noise, can be described by Gaussian distributions and can be seen as Gaussian noise [15,16], but traditional demodulation algorithms, such as the cross-correlation one, cannot suppress the influence of Gaussian noise [17]. A brief explanation is given below.

Assuming that the reflection spectra of the reference FBG-FP and the sensing FBG-FP are *x*(*n*) and *y*(*n*), respectively, *x*(*n*) and *y*(*n*), which contain sensing signals and many kinds of noises, are given by the following formulae:

(1) $$x(n)=s(n)+z_{1}(n)$$

(2) $$y(n)=s(n-\tau_{0})+z_{2}(n)$$

where there is a wavelength difference between *s*(*n*) and *s*(*n*−*τ*_0_), *z*_1_(*n*) and *z*_2_(*n*) are the noises including Gaussian noise.

If there are no noises, the cross-correlation of *x*(*n*) and *y*(*n*) will be:

(3) $$R_{xy}(\tau )=R_{ss}(\tau -\tau_{0})$$

When *τ* equals to *τ*_0,_*R_xy_*(*τ*) will be maximal and we can get the wavelength difference by calculating the location of the peak value of *R_xy_*(*τ*). However, there are, in fact, many noises, so the cross-correlation of *x*(*n*) and *y*(*n*) should be:

(4) $$R_{xy}(\tau )=R_{ss}(\tau -\tau_{0})+R_{z_{1}s}(\tau -\tau_{0})+R_{sz_{2}}(\tau )+R_{z_{1}z_{2}}(\tau )$$

As we know that the noises *z*_1_(*n*) and *z*_2_(*n*) are partly correlative, the result of the cross-correlation of *z*_1_(*n*) and *z*_2_(*n*) will not vanish. The noises are irrelevant to sensing signals *x*(*n*) and *y*(*n*), the result of the cross-correlation between sensing signals and noises will not vanished, so we can get the following conclusion:

(5) $$R_{z_{1}s}(\tau -\tau_{0})=0,R_{sz_{2}}(\tau )=0,R_{z_{1}z_{2}}(\tau )\neq 0$$

The location of the peak value of *R_xy_*(*τ*) is affected by *Rz_1_z_2_*(*τ*), so the final demodulation result of the wavelength difference is not accurate due to the subsistent *Rz_1_z_2_*(*τ*). Therefore, an algorithm to solve this problem is very necessary. In particular, there are an amount of Gaussian noises in earthquake monitoring [18]. As we know that the cross third-order cumulant can suppress the Gaussian noise completely and estimate time delays [19], we can use the characteristics of the cross third-order cumulant to solve the influence of relevant Gaussian noises and get a more accurate demodulation result for the wavelength difference.

**Figure 1:**
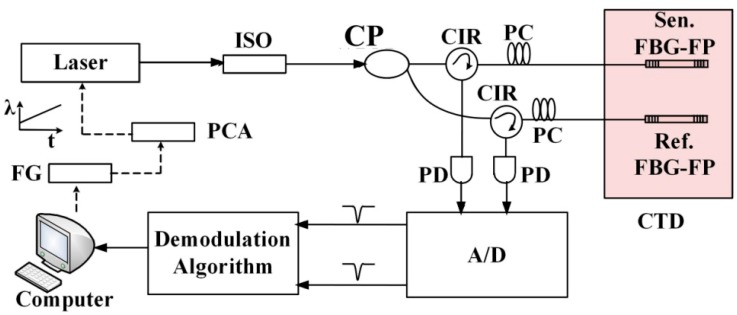
The schematic configuration of the demodulation system. ISO, isolator; CP, coupler; CIR, circulator; PC, polarization controller; PD, photodiode; A/D, analog-to-digital converter; FG, function generator; PCA, piezoelectric ceramic amplifier. CTD, constant temperature device.

This paper presents a method using the Hilbert transform and cross third-order cumulant for demodulation of FBG-FP static-strain sensors, which are used to measure crust deformation. The schematic configuration of the demodulation system is shown in Figure 1. The beam from the tunable fiber laser (NKT, line-width 100 Hz) is spilt into a pair of FBG-FPs, one for strain sensing and the other for compensating the errors due to temperature disturbance and laser wavelength drift. Two polarization controllers are used to eliminate the polarization effects. The strain of the sensing FBG-FP is demodulated by a demodulation algorithm. In this demodulation algorithm, we apply the cross third-order cumulant to cross-correlation processing in noise-contaminated FBG-FP reflection spectra for static-strain measurement. Since the third-order cumulant is insensitive to Gaussian signals, the Gaussian noises are suppressed as well as any correlated Gaussian noises. Meanwhile, the reflection spectrum of FBG-FP also obeys a Gaussian distribution. The Hilbert transform was used to change the Gaussian feature of the FBG-FP’s reflection spectra first, and a higher static-strain resolution than achievable with the traditional cross-correlation method is obtained by using this technique.

## 2. Theory

**Figure 2:**
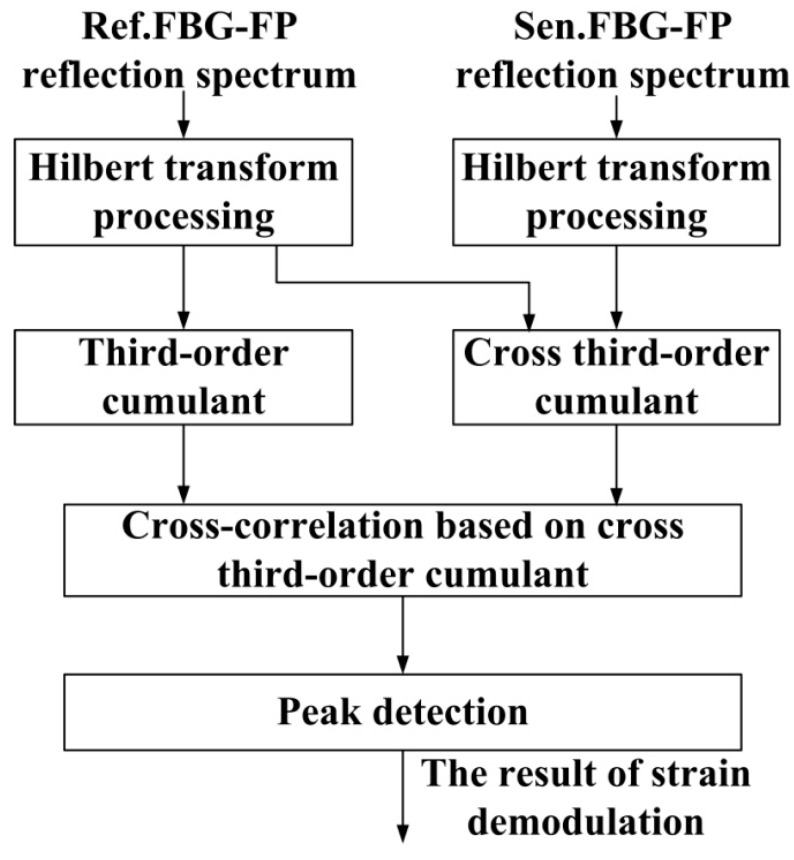
The schematic diagram of the demodulation algorithm based on the Hilbert transform and third-order cumulant.

The schematic diagram of the demodulation algorithm based on the Hilbert transform and third-order cumulant for calculating wavelength differences is shown in Figure 2. This algorithm consists of three steps: (1) apply Hilbert transform processing on the reflection spectra from FBG-FPs to change the Gaussian distribution of sensing signals; (2) calculate the third-order cumulant and the cross third-order cumulant of the two reflection spectra; and (3) calculate the cross-correlation of the third-order cumulant and cross third-order cumulant. Then the wavelength difference between the two sensing signals can be calculated by a peak detection technique and we can get the FBG-FP strain information according to the magnitude of the wavelength difference. A detailed theoretical model is given below.

Assuming a Gaussian random variable is *x*, its mean value is zero and variance is σ^2^, the probability density function of *x*:

(6) $$f(x)=\frac{1}{\sqrt{2\pi \sigma }}\exp(-\frac{x^{2}}{2\sigma^{2}})$$

As we know, the high-order cumulant of Gaussian random variable is identically equal to zero [19], so the high-order cumulant of Gaussian signals can eliminate the influence of the Gaussian noise completely in theory.

Because of the curves of FBG-FP’s reflectance spectra obey the Gaussian distribution, the first step of the demodulation algorithm is to change the Gaussian distribution characteristica of the reflectance spectra. Here we apply the Hilbert transform to do such processing. The Hilbert transform can change the valley of reflectance spectra to zero. The definition of the Hilbert transform can be shown by:

(7) $$X(t)=H[x(t)]=\frac{1}{\pi }\int \frac{x(t-\xi )}{\xi }d\xi$$

where *x*(*t*) is the original signal, *X*(t) is the Hilbert transform of *x*(*t*). Through the Hilbert transform, the reflectance spectrum of the referencing FBG-FP is transformed into *X*(*t*). In the same way, the reflectance spectrum of the sensing FBG-FP can be transformed into *Y*(*t*). The Hilbert transform does not change the wavelength difference of the two reflectance spectra.

After Hilbert transform processing, the cross third-order cumulant is used to process the result of the Hilbert transform and get a group of noise-removed signals which can be used to calculate the wavelength difference of the two FBG-FPs accurately by the cross-correlation method. Here, assuming the *X*(*n*) and *Y*(*n*) are shown as:

(8) $$X(n)=S(n)+W_{1}(n)$$

(9) $$Y(n)=S(n-\tau_{0})+W_{2}(n)$$

where the wavelength difference between *S*(*n*) and *S*(*n-τ*_0_) is equal to the wavelength difference between *s*(*n*) and *s*(*n−τ*_0_) in Equations (1) and (2). *W*_1_(*n*) and *W*_2_(*n*) are noises.

As we know the Gaussian noise source is a zero-mean stationary random process. The third-order cumulant of *X*(*n*) is noted as *C_XXX_*(*τ*, *ρ*), and the cross third-order cumulant of *X*(*n*) and *Y*(*n*) is noted as *C_XYX_*(*τ*, *ρ*) [20]:

(10) $$C_{XXX}(\tau ,\rho )=cum[S(n)+W_{1}(n),S(n+\tau )+W_{1}(n+\tau ),S(n+\rho )+W_{1}(n+\rho )]$$

(11) $$C_{XYX}(\tau ,\rho )=cum[S(n)+W_{1}(n),S(n+\tau -\tau_{0})+W_{1}(n+\tau ),S(n+\rho )+W_{1}(n+\rho )]$$

As the third-order cumulant of Gaussian noise is zero, we can get:

(12) $$C_{XXX}(\tau ,\rho )=R_{sss}(\tau ,\rho )$$

(13) $$C_{XYX}(\tau ,\rho )=R_{sss}(\tau -\tau_{0},\rho )$$

From Equations (12) and (13), we know that the Gaussian noises are completely suppressed. Then we take one-dimensional slice of the third-order cumulant and cross third-order cumulant when *ρ* is equal to zero:

(14) $$C_{XXX}(\tau )=C_{XXX}(\tau ,0)=\frac{1}{N}\sum_{n=0}^{N-1}X(n)X(n+\tau )X(n)$$

(15) $$C_{XYX}(\tau )=C_{XYX}(\tau ,0)=\frac{1}{N}\sum_{n=0}^{N-1}X(n)Y(n+\tau )X(n)$$

From Equations (14) and (15), we know that *C_XXX_*(*τ*) and *C_XYX_*(*τ*) are two processed signals without Gaussian noises but containing the information of the wavelength difference of the two FBG-FPs’ reflection spectra.

Then *C_XXX_*(*τ*) and *C_XYX_*(*τ*) can be used to calculate the wavelength difference by a cross-correlation method without the influence of Gaussian noises. The cross-correlation of *C_XXX_*(*τ*) and *C_XYX_*(*τ*) is shown below:

(16) $$R(\tau )=IFFT\left[\frac{FFT\left[C_{XXX}(\tau )\right]\cdot FFT\left[C_{XYX}(\tau )\right]}{N}\right]$$

Finally, we take the location of peak value of *R*(*τ*) as an estimate of the wavelength difference*τ*_0_ by peak detection technique.

## 3. Simulation Analysis

In order to verify the precision and feasibility of the demodulation algorithm, a simulation analysis is presented below. The original sensing signals *x*(*n*) and *y*(*n*) are sampled in the laboratory in a quiet environment. The term *x*(*n*) is obtained by using a tunable fiber laser to sweep FBG-FP 1 as shown in Figure 1, and *y*(*n*) is obtained by shifting *x*(*n*) 510 sampling points, so the actual wavelength difference of *x*(*n*) and *y*(*n*) is 510 sampling points.

**Figure 3:**
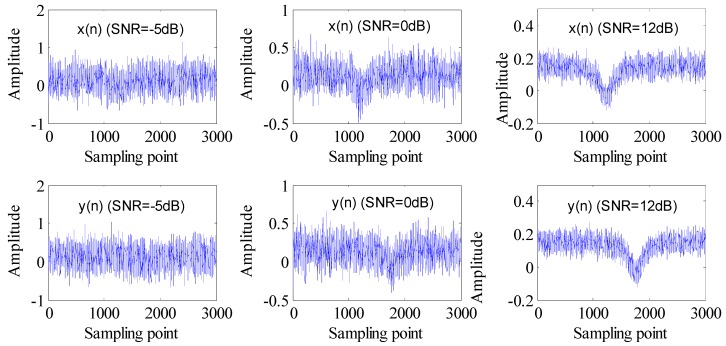
The signals *x*(*n*) and *y*(*n*) with SNR of −5 dB, 0 dB, 12 dB.

The reflectance spectra of referencing FBG-FP *x*(*n*) and sensing FBG-FP *y*(*n*) with SNR of −5 dB, 0 dB and 12 dB are shown in Figure 3, respectively. From Figure 3, we can find that the original signals are submerged in noises when SNR is low. Obviously, the noises, including the relevant Gaussian noise will affect the precision of demodulation for the wavelength difference of two FBG-FPs’ reflectance spectra. An algorithm to solve this problem is very necessary.

**Figure 4:**
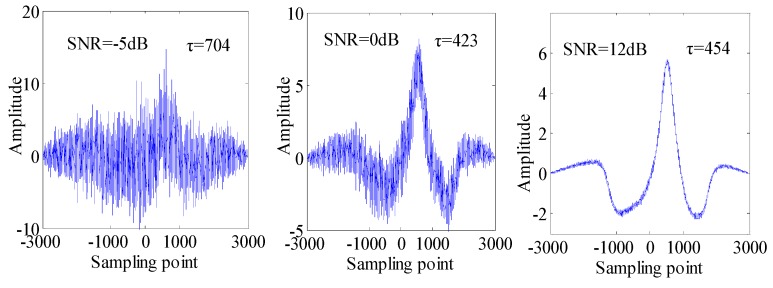
The cross-correlation of *x*(*n*) and *y*(*n*) under SNR = −5 dB, 0 dB, 12 dB.

To begin with, we can dispose the results of traditional cross-correlation with different SNR. The Figure 4 shows the simulation of the cross-correlation of *x*(*n*) and *y*(*n*) with SNR of −5 dB, 0 dB, 12 dB. The simulation value τ of wavelength difference in Figure 4 is 704, 423, and 454 sampling points, respectively. We can find that the peak location of the cross-correlation is closer to the true wavelength difference when the SNR of the sensing signals increases, and a low SNR may cause a large deviation between τ and the true wavelength difference.

**Figure 5:**
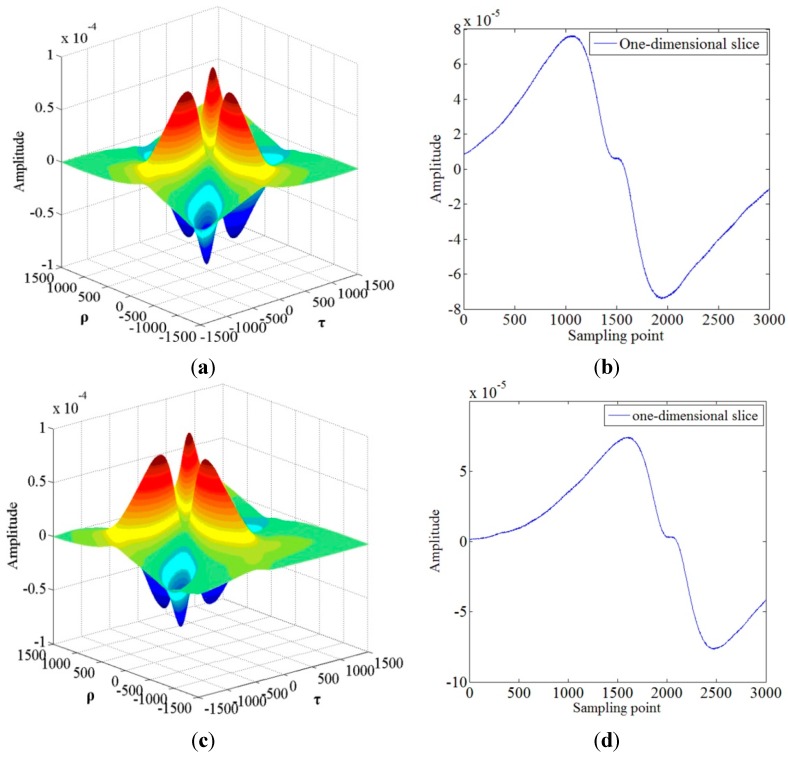
The cross third-order cumulant curves and one-dimensional slice: (**a**) is the third-order cumulant of *X*(*n*); (**b**) is one-dimensional slice of (a); (**c**) is the cross third-order cumulant of *X*(*n*) and *Y*(*n*); (**d**) is one-dimensional slice of (c).

For the cross third-order method, the Hilbert transform should be used for changing the Gaussian distribution of the reflection spectra from the two FBG-FPs first. Then the cross third-order cumulant is used to utilize the result of the Hilbert transform and get a group of noise-free signals which can be used to calculate the wavelength difference of the two FBG-FPs. As the theoretical model described, the third-order cumulant of *X*(*n*) and it’s one-dimensional slice *C_XXX_*(*τ*), the cross third-order cumulant of *X*(*n*) and *Y*(*n*) and it’s one-dimensional slice *C_XYX_*(*τ*) are shown in Figure 5. The one-dimensional slices of the third-order cumulants are very smooth, implying that the Gaussian noise is suppressed effectively and the two one-dimensional slices contain the wavelength difference information of the reflection spectra from the two FBG-FPs, so we can calculate the wavelength difference *τ* by the cross-correlation method in the next step.

**Figure 6:**
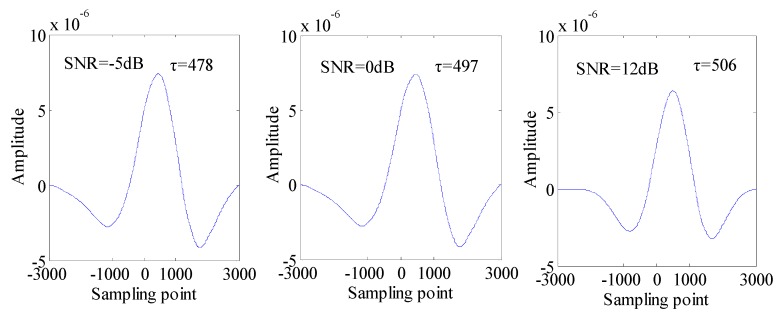
The cross-correlation of *C_XXX_*(*τ*, 0) and *C_XYX_*(*τ*, 0) with SNR of −5 dB, 0 dB, 12 dB.

The cross-correlation results of *C_XXX_*(*τ*) and *C_XYX_*(*τ*) when using the cross third-order cumulant method algorithm are shown in Figure 6. We can see that the simulation value *τ* with SNR of −5 dB, 0 dB, 12 dB is 478, 497, 506 points, respectively. Compared with traditional cross-correlation, the *τ* of the cross-correlation based on the cross third-order cumulant method is more accurate and closer to the actual value. In particular, the computational accuracy of *τ* will be greatly improved when so many relevant Gaussian noises exist and the SNR of sensing signals is low.

**Figure 7:**
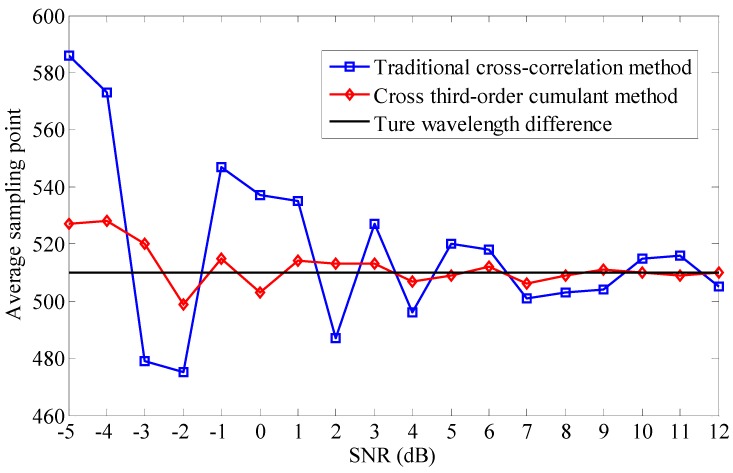
The simulation average values *τ* with the two different methods at different SNR levels.

In addition, 50 pairs of sensing signals with random relative Gaussian noise SNR level from −5 dB to 12 dB are used for proving our conclusion. The traditional cross-correlation method and cross third-order cumulant method are used to deal with these data, respectively, and an average processing of cross-correlation results is made at each SNR level. The simulation average values *τ* with the two different methods are shown in Figure 7. We can find that the estimation value *τ* by the cross third-order cumulant method is more accurate with respect to the actual wavelength difference than the traditional cross-correlation method whether the SNR of the sensing signals is high or low. Especially, the traditional cross-correlation method fails to work well when the SNR is less than −3 dB, but the cross third-order cumulant method presents a relatively high precision in this case.

**Figure 8:**
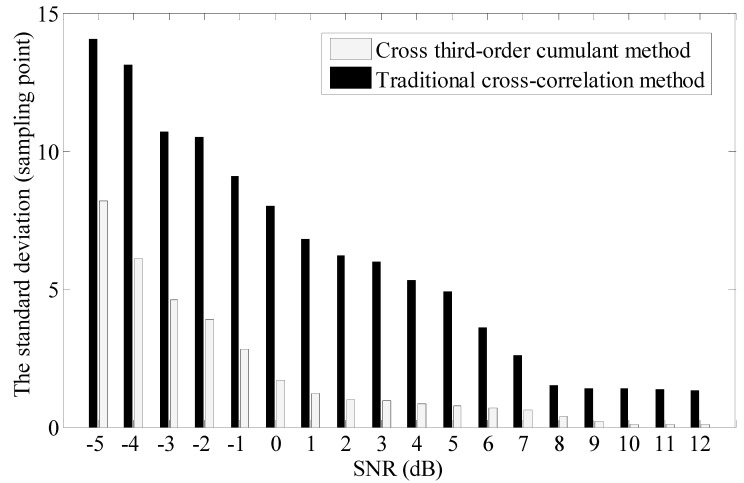
The standard deviation of different algorithms with different SNR.

In order to verify the correctness of the simulation algorithm, other tests are also done under the condition of different sampling shifts. We can find that the proposed algorithm has a higher demodulation accuracy than the traditional cross-correlation method and shows similar characteristics when the simulation is tested based on different sampling shifts. The authors have given the standard deviation of different algorithms with different SNR as shown in Figure 8. The results show that the proposed method has more advantages than the traditional method. The standard deviation of the proposed algorithm is less than 1 sampling point.

## 4. Experiments and Discussion

**Figure 9:**
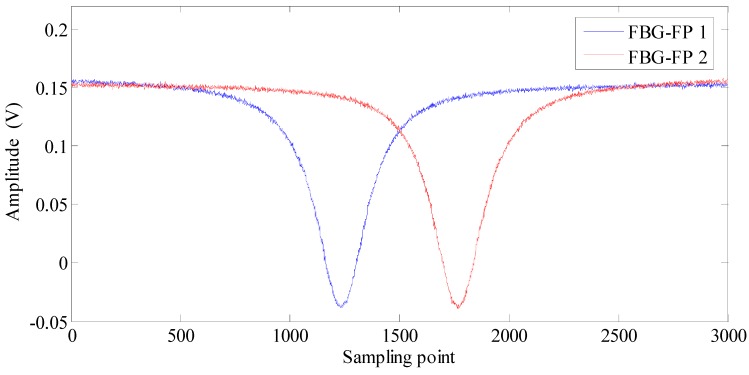
A group of reflection spectrums of FBG-FPs in the laboratory.

In the laboratory experiments, two FBG-FPs work as a strain sensing head and a reference sensing head, respectively. The two sensors have the same technical parameters such as nominal center wavelength 1549.720 nm, 3 dB bandwidth of 0.22 nm and peak reflectivity 99.5%. The free spectral range (FSR) and the bandwidth of the FBG-FPs are 4.1 pm and 0.9 MHz, respectively. We use a triangular wave to drive the tunable fiber laser (NKT, linewidth 100 Hz). The period of the triangular waveform is ten seconds, thus the laser sweeping period is ten seconds and the tunable range of the laser source is 4 pm. The sampling frequency is 10 kHz and there are about 100,000 sampling points during one sweeping period. A group of reflection spectrums of FBG-FPs during one wavelength sweep period are shown in Figure 9.

**Figure 10:**
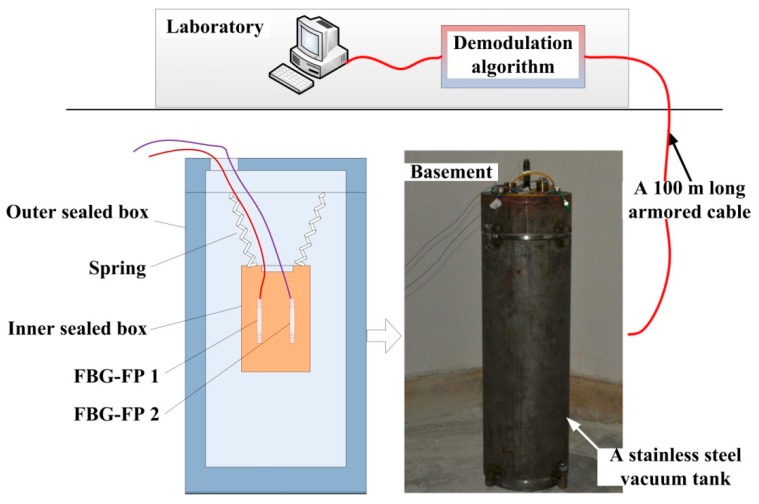
The scheme of laboratory experiments.

In order to verify the ultimate strain resolution of the proposed algorithm, a quiet environment and free strain conditions should be ensured. In our experiment configuration, the two FBG-FPs are packaged in a double sealed box and the inner sealed box is hanging by a spring to suppress any low-frequency vibrations. The sealed box is put into 10-cm thick stainless steel outer sealed tank which is used to suppress environmental noise interference and maintain a relatively constant temperature (shown in Figure 10). Then we put the stainless steel tank in the 5 m deep basement where the environment is quiet and the temperature is relatively constant. The sensing FBG-FPs and reference FBG-FP are connected to the demodulation system by a 100 m long armored optical cable. As shown in Figure 9, the SNR of the reflection spectrums of FBG-FPs in the laboratory is greater than 30 because of the favorable experimental environment.

**Figure 11:**
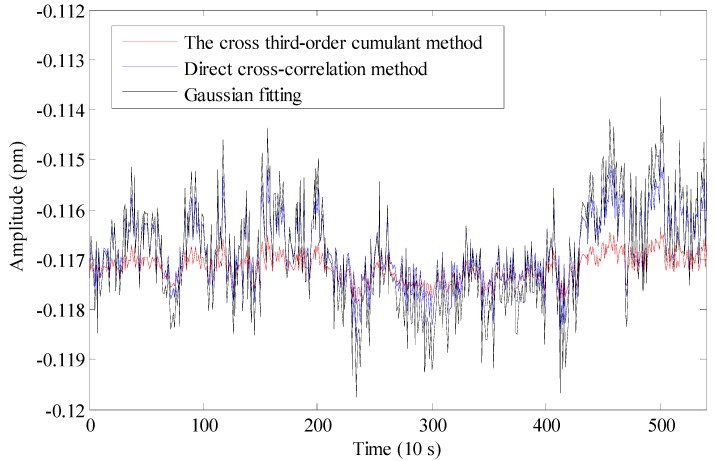
The demodulation results of different methods.

The proposed Hilbert transform and cross third-order cumulant method are implemented in a Field Programmable Gate Array (FPGA) board card (NI 7966R). The proposed algorithm takes up more logic resources and multipliers of the FPGA board card than traditional cross-correlation method and Gaussian fitting method. The FPGA resource occupation rates of the proposed method, traditional cross-correlation method and Gaussian fitting method respectively are 92%, 47%, 44%. A proper logical code design is very important for our proposed algorithm. The demodulation result by the proposed cross third-order cumulant method is compared with traditional cross-correlation method and Gaussian fitting method, which are shown in Figure 11. It shows that the three kinds of results have the same strain trend and the cross third-order cumulant method presents a higher accuracy. At the same time, this system shows a characteristic of long-term stability, which is important for long-term strain monitoring in the geophysical field.

Considering the strain sensitivity of 1.2 pm/με of FBG-FP approximately, we can see that the static-strain resolution of the proposed demodulation algorithm is 0.9 nε (about 0.00108 pm standard deviation), while the resolution of the traditional cross-correlation algorithm and the Gaussian fitting algorithm are 3.5 nε (about 0.0042 pm standard deviation) and 5.6 nε (about 0.0067 pm standard deviation), respectively, which are shown in Table 1. These results indicate that the cross third-order method is more robust to correlated noise and less sensitive to non-stationary data because of its unique higher order statistics features. Compared with the previous simulation, the experimental results and the simulation results are consistent, showing that the cross third-order method is accurate. The static-strain resolution of this system is now mainly limited by the nonlinearity of the voltage-wavelength response during the wavelength sweeping.

**Table 1:** The demodulation resolutions of different algorithms.

Methods	Standard Deviation (nε)
Hilbert transform and third-order cumulant method	0.9
Cross-correlation method	3.5
Gaussian fitting method	5.6

This paper, as well as the authors’ previous paper [13], proposes a sealed box and a 10-cm thick stainless steel tank for suppressing environmental noise interference and maintaining a relatively constant temperature. The actual SNR of the reflection spectra of FBG-FPs in the laboratory is greater than 30, so the amount of the enhancement in the strain resolution of the proposed algorithm is not obvious compared with the previous method, but there is always a lot of noise including random vibration and acoustic disturbances from actual sensing environments. As a result, the SNR of the reflection spectra of FBG-FPs may be smaller than 1. In this case, the proposed algorithm will show a significant advantage compared with the traditional cross-correlation method and the method in reference [13]. In their future work, the authors will make a more in-depth analysis on the mathematical model of the low-SNR reflection spectra of FBG-FPs and try to further improve the demodulation precision of low-SNR strain signals.

## 5. Conclusions

This paper presents a high-resolution demodulation algorithm based on the Hilbert transform and cross third-order cumulant for FBG-FP static-strain sensors. A detailed theory model and a simulation analysis are also presented. In our algorithm configuration, the Gaussian distribution of the reflection spectra of two sensing signals is changed via the Hilbert transform first. Then the third-order cumulant can be used to suppress the relevant Gaussian noises. By using this method, we successfully solve the problem that the static-strain demodulation resolution of FBG-FP sensors is seriously influenced by relevant Gaussian noises when using the traditional cross-correlation method. A higher static-strain resolution of 0.9 nε is obtained compared to the traditional cross-correlation method in the laboratory environment. The intrinsic detection principle of FBG and FBG-FP sensors is based on the relationship between the strain of the Bragg grating and the sensed physical quantities (such as deformation, acceleration, acoustic pressure, and so on). The proposed demodulation algorithm and hardware system are designed for deformation monitoring applications. Such a high infrasonic strain resolution is also very suitable for elastic wave detection in the field of natural earthquake monitoring, ultra-low frequency acoustic monitoring, and so on.
